# Arthrocentesis Plus Injectable Platelet-Rich Fibrin in Patients With Temporomandibular Joint Dysfunction: An Updated Meta-Analysis With Trial Sequential Analysis

**DOI:** 10.7759/cureus.110871

**Published:** 2026-06-15

**Authors:** Maryam Bader Alzamanan, Dhari Mohammed Alhajri, Fay Hamed Alduaij, Khaled Walled Abdullah, Mohammad Homoud Alnoumas, Yousef Abdulghafour, Athoub Almousawi, Maryam Nasser Alshammari, Sarah Alajmy

**Affiliations:** 1 Department of Dentistry, Kuwait Ministry of Health, Kuwait City, KWT; 2 Department of Dentistry, Qortuba Health Center, Kuwait City, KWT; 3 Department of Dentistry, Al-Adan Primary Healthcare Center, Al-Adan, KWT; 4 Department of Dentistry, West Subahiya Polyclinic, Kuwait Ministry of Health, Subahiya, KWT; 5 Department of Dentistry, Al-Adan Hospital, Kuwait Ministry of Health, Hadiya, KWT

**Keywords:** arthrocentesis, injectable platelet-rich fibrin (i-prf), systematic review and meta-analysis, temporomandibular joint dysfunction, tmd

## Abstract

Temporomandibular joint (TMJ) dysfunction (TMD) is a common disorder associated with chronic pain, impaired mandibular movement, and reduced quality of life (QoL). Arthrocentesis is widely used as a minimally invasive treatment for symptomatic TMD; however, incomplete symptom resolution remains common. Injectable platelet-rich fibrin (i-PRF) has recently emerged as a biologic adjunctive therapy with potential regenerative and anti-inflammatory effects. Therefore, we conducted an updated systematic review and meta-analysis to evaluate the efficacy of arthrocentesis combined with i-PRF in patients with TMD. A comprehensive literature search was performed using PubMed, Scopus, Web of Science (WoS), and Cochrane Central Register of Controlled Trials (CENTRAL) from inception to May 1, 2026. Randomized controlled trials (RCTs) comparing arthrocentesis plus i-PRF with control interventions were included. The primary outcome was the change in pain score measured using the Visual Analogue Scale (VAS). Secondary outcomes included maximum mouth opening (MMO), lateral excursion, protrusive movement, and QoL. Pooled estimates were calculated using a random-effects model and expressed as mean differences (MDs) with 95% confidence intervals (CIs). Nine RCTs involving 320 patients (374 joints) were included. Arthrocentesis combined with i-PRF significantly reduced pain scores compared with control interventions (MD -1.62, 95% CI: -2.28 to -0.96; p<0.001; I^2^= 73.55%). TSA confirmed the conclusive and sufficient confidence levels of the results. Significant improvements were also observed in MMO (MD 5.03 mm, 95% CI: 2.99 to 7.07; p<0.001; I^2^= 84.87), contralateral excursion (MD 0.94 mm, 95% CI: 0.41 to 1.48; p<0.001), and protrusive movement (MD 1.08 mm, 95% CI: 0.48 to 1.69; p<0.001). No significant differences were observed in right or left lateral excursion or overall QoL. Arthrocentesis combined with i-PRF appears to significantly improve pain and mandibular functional outcomes in patients with TMD. The available evidence supports i-PRF as a promising adjunctive minimally invasive therapy; however, larger RCTs with longer follow-up durations are warranted to confirm long-term efficacy and establish standardized treatment protocols.

## Introduction and background

Temporomandibular joint (TMJ) dysfunction (TMD) represents one of the most common disorders affecting the craniofacial region and is frequently associated with chronic pain, limited mandibular movement, joint sounds, and impaired quality of life (QoL) [1]. The condition encompasses a broad spectrum of pathological entities, including internal derangement, disc displacement, and degenerative joint disease, which may substantially interfere with daily activities such as chewing, speaking, and mouth opening [2,3].

Arthrocentesis has emerged as a widely accepted minimally invasive procedure for the management of symptomatic TMD, particularly in patients with internal derangement or osteoarthritis refractory to conservative treatment [4]. However, despite these benefits, symptom recurrence and incomplete functional recovery remain challenges in some patients, prompting growing interest in adjunctive biologic therapies that may enhance tissue healing and prolong clinical improvement.

Injectable platelet-rich fibrin (i-PRF) is a second-generation autologous platelet concentrate that has recently gained attention in regenerative medicine and oral-maxillofacial surgery due to its high concentration of platelets, leukocytes, fibrin matrix, and growth factors [5,6]. Compared with conventional platelet-rich plasma, i-PRF is prepared without anticoagulants and demonstrates a sustained release of bioactive mediators involved in angiogenesis, anti-inflammatory modulation, and tissue regeneration [7]. In the context of TMD, intra-articular i-PRF injection following arthrocentesis has been proposed to improve pain control, enhance cartilage repair, and restore joint function [8].

Previous systematic reviews and meta-analyses have suggested that platelet concentrates used as adjuncts to arthrocentesis may improve pain intensity, maximum mouth opening (MMO), and functional outcomes in patients with TMD [7,8]. However, the available evidence remains limited by the small number of randomized controlled trials (RCTs), heterogeneity in platelet concentrate preparation protocols, variations in follow-up durations, and inconsistent outcome reporting. Furthermore, the specific effectiveness of i-PRF following arthrocentesis has not been comprehensively synthesized with consideration of different follow-up periods and pain-related outcomes.

Therefore, we conducted an updated systematic review and meta-analysis of RCTs to evaluate the effectiveness of arthrocentesis plus i-PRF in patients with TMD. We hypothesized that arthrocentesis combined with i-PRF would result in greater improvements in pain reduction and mandibular function compared with arthrocentesis alone.

## Review

Methods

This systematic review and meta-analysis was conducted according to the Preferred Reporting Items for Systematic Reviews and Meta-Analysis (PRISMA) guidelines [9] and the recommendations of the Cochrane Handbook for Systematic Review of Interventions [10].

Literature Search

A comprehensive literature search was performed using four electronic databases, including PubMed, Scopus, Web of Science (WoS), and Cochrane Central Register of Controlled Trials (CENTRAL) from inception to May 1, 2026. The search strategy included combinations of the following keywords and medical subject headings (MeSH) terms: “temporomandibular joint disorders”, “temporomandibular joint disc”, “osteoarthritis”, “arthropathy”, “arthrocentesis”, and “injectable platelet-rich fibrin”. Detailed database-specific search strategies are provided in Table 1. Reference lists of relevant articles were manually screened for backward citation analysis to identify additional eligible studies. No restrictions were applied based on publication status, except for an English filter.

**Table 1:** Detailed search strategy for each database. CENTRAL: Central Register of Controlled Trials

Database	Keywords	Filter	Results
PubMed	(((Temporomandibular Joint Disorders) OR Temporomandibular Joint Disc) OR (temporomandibular AND (joint OR TMJ OR temporo-mandibular) AND (disorder OR disease OR pain OR dysfunction OR internal derangement OR disc displacement OR disk displacement OR osteoarthritis OR arthropathy))) AND (Arthrocentesis OR arthrocentesis OR joint lavage OR TMJ lavage OR temporomandibular lavage) AND (Platelet-Rich Fibrin OR platelet-rich fibrin OR platelet rich fibrin OR PRF OR i-PRF OR iPRF OR injectable platelet-rich fibrin OR liquid platelet-rich fibrin OR injectable PRF)	English language	N = 39
Scopus	TITLE-ABS-KEY((temporomandibular OR "temporo-mandibular") AND (joint OR "TMJ") AND (disorder OR disease OR pain OR dysfunction OR "internal derangement" OR "disc displacement" OR "disk displacement" OR osteoarthritis OR arthropathy)) AND TITLE-ABS-KEY(arthrocentesis OR "joint lavage" OR "TMJ lavage" OR "temporomandibular lavage") AND TITLE-ABS-KEY("platelet-rich fibrin" OR "platelet rich fibrin" OR "PRF" OR "i-PRF" OR "iPRF" OR "injectable platelet-rich fibrin" OR "liquid platelet-rich fibrin" OR "injectable PRF")	English language	N = 52
Web of Science	TS=((temporomandibular OR "temporo-mandibular") AND (joint OR TMJ) AND (disorder OR disease OR pain OR dysfunction OR "internal derangement" OR "disc displacement" OR "disk displacement" OR osteoarthritis OR arthropathy)) AND TS=(arthrocentesis OR "joint lavage" OR "TMJ lavage" OR "temporomandibular lavage") AND TS=("platelet-rich fibrin" OR "platelet rich fibrin" OR PRF OR "i-PRF" OR iPRF OR "injectable platelet-rich fibrin" OR "liquid platelet-rich fibrin" OR "injectable PRF")	English language	N = 41
Cochrane CENTRAL	(((Temporomandibular Joint Disorders) OR Temporomandibular Joint Disc) OR (temporomandibular AND (joint OR TMJ OR temporo-mandibular) AND (disorder OR disease OR pain OR dysfunction OR internal derangement OR disc displacement OR disk displacement OR osteoarthritis OR arthropathy))) AND (Arthrocentesis OR arthrocentesis OR joint lavage OR TMJ lavage OR temporomandibular lavage) AND (Platelet-Rich Fibrin OR platelet-rich fibrin OR platelet rich fibrin OR PRF OR i-PRF OR iPRF OR injectable platelet-rich fibrin OR liquid platelet-rich fibrin OR injectable PRF)	English language	N = 19

Eligibility Criteria and Screening

Studies were included if they met the prespecified Population, Intervention, Comparison, Outcome (PICO) criteria [11], as shown in Table 2. After removal of duplicate records, two reviewers independently screened titles and abstracts for relevance. Full-text articles were subsequently assessed for eligibility based on the predefined inclusion and exclusion criteria. Any disagreements during the screening process were resolved through discussion until consensus was achieved.

**Table 2:** Inclusion and exclusion criteria. TMD: temporomandibular joint dysfunction; i-PRF: injectable platelet‑rich fibrin

Criterion type	Domain	Description
Inclusion	Population	Patients diagnosed with symptomatic TMD internal derangement
	Intervention	Arthrocentesis of the upper joint space (lavage with Ringer’s lactate or saline) followed by intra‑articular injection of i‑PRF
	Comparator	Arthrocentesis alone or conservative/no active treatment
	Outcome	Reported at least one predefined outcome of interest
Exclusion	Study design	Observational studies
		Review articles
	Publication type	Conference abstracts without full text

Endpoints

The primary outcome was the change in pain score from baseline to the end of follow-up, measured using the Visual Analogue Scale (VAS) [12]. The secondary outcomes were the mean change of MMO, contralateral, right, and left lateral excursion, protrusive movement, and QoL using Oral Health Impact Profile-14 (OHIP-14) [13].

Quality Assessment

The methodological quality of the included RCTs was independently assessed by two reviewers using the Cochrane Risk of Bias 2 (ROB-2) tool, which evaluates bias arising from the randomization process, deviations from intended interventions, missing outcome data, outcome measurement, and selection of the reported result [14]. Any discrepancies in quality assessment were resolved by consensus.

Data Extraction

Data extraction was performed independently by two reviewers using a standardized extraction Excel form (Microsoft Corp., Redmond, USA). Extracted data included: (i) study characteristics such as study identification (ID), country, design, population, total sample size, intervention and comparator arms, inclusion and exclusion criteria, measured outcomes, follow-up duration, and key findings; (ii) patients characteristics such as age, sex, laterality, Wilkes stage, symptoms duration, baseline pain, and baseline MMO; (iii) risk of bias domains; and (iv) outcomes measures.

Statistical Analysis

Meta-analysis was performed when outcomes were reported by at least two studies with comparable measures. Mean changes and corresponding standard deviations (SDs) were calculated as the difference between baseline and follow-up values. Continuous outcomes were pooled using mean difference (MD) with corresponding 95% confidence intervals (CIs). A random-effects model was applied to account for anticipated clinical and methodological heterogeneity across studies. Statistical heterogeneity was assessed using the I^2^ statistic, with values >50% considered indicative of substantial heterogeneity.

Furthermore, we performed a leave-one-out sensitivity analysis to assess the influence of individual studies on the overall pooled results, a digital object identifier (DOI) plot with the Luis Furuya-Kanamori (LFK) index [15] to detect the asymmetry between the included studies, and a subgroup analysis based on follow-up duration (three, six, and 9-12 months) for the studied outcomes. Additionally, we explored the change in pain scores during chewing, jaw movements, and palpation as a subgroup-separated analysis from the primary outcome.

Additionally, a trial sequential analysis (TSA) was performed for the primary outcome to assess whether the evidence generated from the analysis was reliable and conclusive. The level of confidence was considered conclusive and sufficient, indicating no other studies were needed, when the Z-curve of the TSA line crossed both the conventional boundary and the sequence monitoring boundary. Conversely, if the Z-curve did not cross any boundary on the curve, the evidence was considered not conclusive, indicating further trials were required. In this meta-analysis, we used an alpha error of 0.05 and a statistical power of 95% (β = 0.05).

We used the Grading of Recommendations Assessment, Development, and Evaluation (GRADE) scale [16] to evaluate the strength and level of evidence for recommendations stratified as follows: high quality, indicating no further research was needed and that it was unlikely to change the confidence of the effects estimations; moderate quality, indicating that further studies may affect the confidence of the effects estimation; low quality, indicating further research is likely to have a crucial impact on the confidence of the effects estimation and may change the estimation; and very low quality, indicating that cannot be certain about this estimation. Furthermore, we performed random-effect meta-regression between the effect size of pain and baseline MMO.

All statistical analyses were performed using Stata/MP 19 software (StataCorp LLC, College Station, USA), and statistical significance was defined as a two-sided p-value of less than 0.05.

Results

Literature Review and Screening

**Figure 1:**
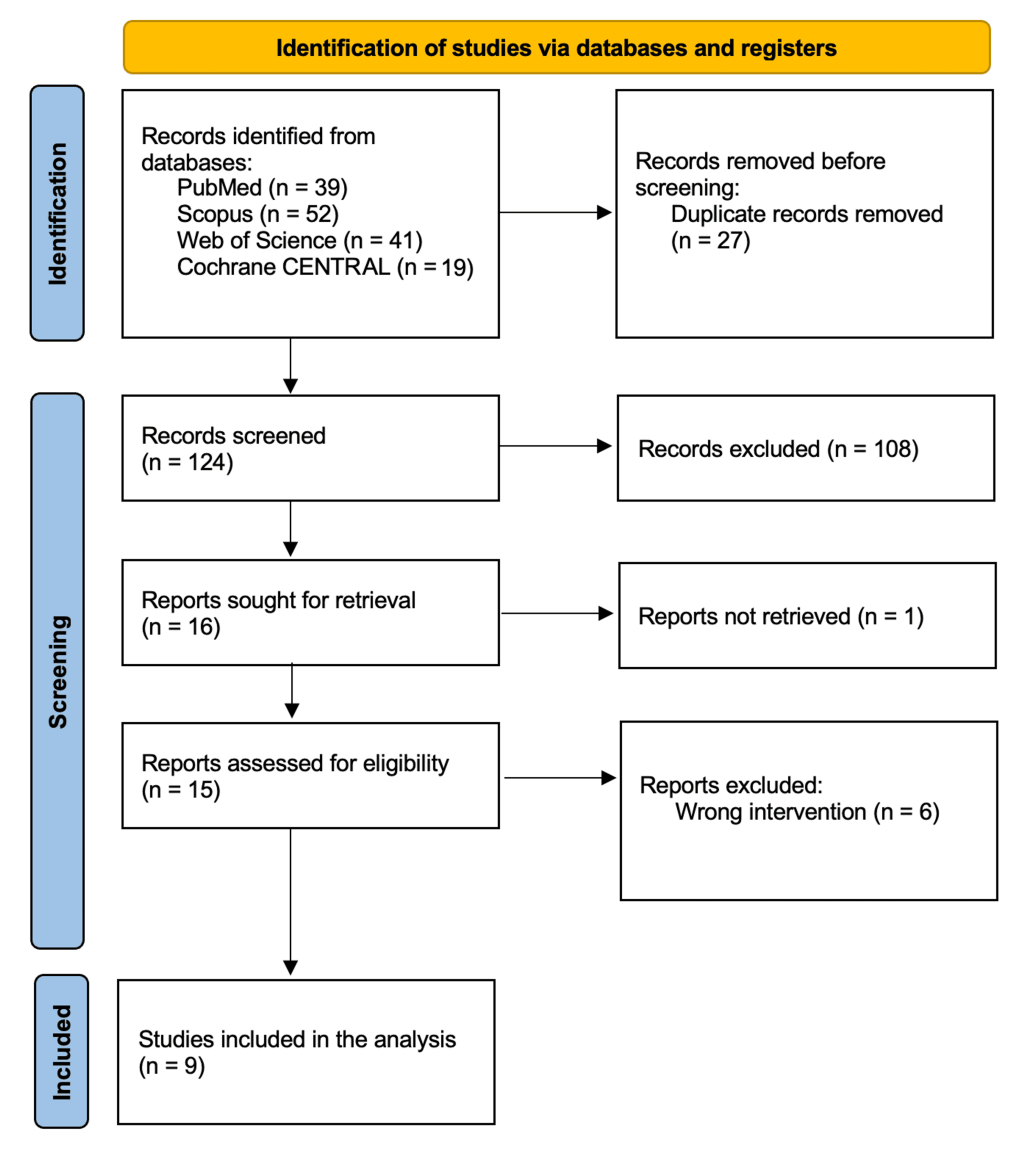
PRISMA flow chart and the selection process. PRISMA: Preferred Reporting Items for Systematic Reviews and Meta-Analysis; CENTRAL: Central Register of Controlled Trials

Our comprehensive literature search identified 151 records across all databases. After the removal of 27 duplicates, 124 articles remained for title and abstract screening. Around 108 citations were excluded at this stage, and one full-text article could not be retrieved. The remaining 15 articles underwent full-text assessment, of which nine studies met the eligibility criteria and were included in the final quantitative analysis [17-25]. The study selection process is illustrated as a PRISMA flow diagram in Figure 1.

Study Characteristics and Quality Assessment

A total of nine RCTs involving 320 patients (374 joints) were included. The studies were published between 2021 and 2026 and were conducted across five countries: Turkey [19-21,25], Egypt [18,22], India [17,24], and Brazil [23]. All trials employed a parallel-group design; seven compared two arms, while two studies incorporated three arms, with follow‑up ranging from three to 12 months. Detailed study characteristics are summarized in Table 3. The RCTs compared arthrocentesis alone, i‑PRF alone, or arthrocentesis combined with i‑PRF in patients with TMJ internal derangement (Wilkes I-V), disc displacement with or without reduction, or osteoarthritis. Sample sizes ranged from 14 to 76 participants, with consistent female predominance (64-93%). Baseline pain scores (VAS 5.9-8.3) and mouth opening (25-42 mm) were comparable across arms. Detailed baseline characteristics of the included patients are stated in Table 4.

**Table 3:** Summary characteristics of the included studies. AC: arthrocentesis; i‑PRF: injectable platelet‑rich fibrin; C‑PRF: concentrated platelet‑rich fibrin; PRP: platelet‑rich plasma; ARS: anterior repositioning splint; MMO: maximum mouth opening; MIO: maximum interincisal opening; VAS: Visual Analogue Scale; OHIP‑14: Oral Health Impact Profile‑14; MFIQ: Mandibular Function Impairment Questionnaire; DC/TMD: Diagnostic Criteria for Temporomandibular Disorders; RCT: randomized controlled trial; TMJ: temporomandibular joint; IPD: individual participant data; QoL: quality of life; CBCT: cone-beam computed tomography; Rx: radiographic examination; DDWoR: disc displacement without reduction; ARS: anterior repositioning splint; TMD: temporomandibular dysfunction; MRI: magnetic resonance imaging; ASA: American Society of Anesthesiologists Physical Status; d: days; wk: weeks; mo: months; y: years

Study	Country	Design	Population	N	Intervention	Comparator	Inclusion criteria	Exclusion criteria	Measured outcomes (primary in bold)	Follow‑up	Key findings
Chaulagain 2025 [17]	India	Single-centre, parallel‑group RCT	Unilateral TMJ intra‑articular pain/dysfunction (Wilkes II-IV) confirmed by MRI; mean age 27-36 y; ≥68% female	32	(1) AC alone; (2) AC + i‑PRF	AC alone	Age 18-50 y; unilateral IPD; Wilkes II-IV on MRI	Age <18 or >50 y; autoimmune disease; MRI contraindication; previous TMJ surgery; congenital/developmental TMJ disorders; TMJ lesions	Pain (VAS) at 3 mo; MIO; lateral and protrusive movements; muscle tenderness (VAS); QoL (OHIP‑14)	Baseline, 10 d, 1 mo, 3 mo	AC + i‑PRF significantly reduced pain and improved MIO and QoL compared with AC or i‑PRF alone at 3 mo. i‑PRF alone was not superior to AC.
Ghoneim 2022 [18]	Egypt	Two-parallel‑group RCT	TMJ disc displacement with reduction (MRI‑confirmed); mean age 27-29 y; 80% female	40	AC + i‑PRF (1.5 mL intra‑articular)	AC alone	Persistent mouth opening restriction; TMJ pain and clicking; failed conservative treatment	Inflammatory/connective tissue disease; neurologic disorders; bony/fibrous ankylosis; previous TMJ surgery; acute infection; anticoagulants/NSAIDs within 48 h; recent corticosteroid use	Pain (VAS), MIO, lateral excursions (mm), clicking	Baseline, 1 wk, 3 mo, 6 mo	AC + i‑PRF produced significantly greater pain reduction, larger MIO and lateral movement gains, and lower clicking prevalence than AC alone at all follow‑up points through 6 mo.
Isık 2022 [19]	Turkey	Single‑blind RCT	TMJ osteoarthritis (DC/TMD + CBCT); MMO <35 mm; failed conservative Rx; mean age 45 y; >90% female	36	AC + four weekly i‑PRF injections	AC alone	One or both TMJs with OA; MMO <35 mm (with deviation); impeded protrusive/lateral movements; localized joint pain; ≥18 y; platelets ≥150,000/mm³	Systemic/malignant disease; previous TMJ surgery; fibrous/osseous ankylosis; cutaneous/otic/articular infection; edentulous; pregnancy/lactation	Pain (VAS - palpation, chewing, jaw movements); MMO; lateral and protrusive movements	Baseline, 1, 2, 3, 6, 12 mo	i‑PRF after AC gave significantly lower pain and larger jaw movement than AC alone up to 12 mo. In the AC‑only group, benefits started to decay after 6 mo.
Isık 2023 [20]	Turkey	Single‑blind RCT	Disc displacement without reduction (DDWoR - DC/TMD + MRI); failed ≥6 mo conservative Rx; mean age 47 y; 90% female	76	AC + four weekly i‑PRF injections	AC alone	Unilateral or bilateral DDWoR; localized pain; limited mouth opening; impeded lateral/protrusive movements; ≥18 y	Hematologic disorders; congenital/inflammatory/malignant joint disease; prior TMJ surgery; edentulism	Pain (VAS - palpation, chewing, jaw movements); MMO; lateral and protrusive movements; treatment success rate	Baseline, 1, 2, 3, 6, 12 mo	Success rate 100% (i‑PRF) vs 73.7% (AC alone). AC + i‑PRF achieved significantly greater pain relief and jaw motion improvement; benefits were sustained for 12 mo, while AC‑only results declined after 6 mo.
Karadayi 2021 [21]	Turkey	Prospective RCT	Unilateral TMJ internal derangement (Wilkes III-V on MRI/CT); failed conservative Rx; mean age 40 y; 47% female	36	AC + single i‑PRF injection (2 mL)	AC alone	Unilateral internal derangement; localized TMJ pain; Wilkes stage ≥3; failed conservative treatment	Autonomic disease; significant mechanical obstruction; acute capsulitis; benign/malignant TMJ lesions; neurological disorders; coagulopathy; allergy	Pain (VAS); MIO; Helkimo Clinical Dysfunction Score (HCDS)	Baseline, 10 d, 1 mo, 3 mo	AC + i‑PRF improved pain and dysfunction significantly more than AC alone at all follow‑ups. The greatest difference was observed in Wilkes stage III.
Nasef 2024 [22]	Egypt	Two‑parallel‑group RCT	Bilateral disc displacement without reduction (MRI‑confirmed); all patients received an anterior repositioning splint (ARS)	30	ARS + AC + single i‑PRF injection	ARS + AC alone	Bilateral DDWoR; symptomatic	Not explicitly reported (standard TMJ surgery/systemic disease contraindications likely applied)	MMO; right/left lateral excursions; pain (VAS); disc position (MRI)	Baseline, 1 wk, 1, 3, 6 mo	Adding i‑PRF to ARS + AC significantly improved MMO and pain at 3 and 6 mo compared with ARS + AC alone. Disc position on MRI remained unchanged in both groups.
Metello Neves 2026 [23]	Brazil	Double‑blind, placebo‑controlled RCT	Articular TMD with joint sounds and clinical disc displacement (DC/TMD criteria); mean age 52 y; 64% female	36	Three monthly intra‑articular i‑PRF injections (1 mL each; no AC)	Lactated Ringer’s solution (no AC)	≥18 y; articular TMD (joint sounds, DDWR/DDWoR)	Prior TMJ surgery or intra‑articular injection; neuropathic pain; systemic polyarthritis; pregnancy/lactation; anticoagulants	Joint sound intensity (VAS); DDWR & DDWoR presence (DC/TMD); MMO	Baseline, 1, 2, 6 mo	i‑PRF significantly reduced joint sounds (-83%) and resolved DDWR/DDWoR signs compared with control at 6 mo. MMO increased more with i‑PRF (+13% vs +6%).
Sharma 2023 [24]	India	RCT	Internal derangement Wilkes I-V (clinical + MRI); age 20-50 y; 14 patients (28 joints)	14	All had AC once, then 6-monthly intra‑articular injections of i‑PRF	All had AC once, then 6-monthly injections of PRP	Wilkes I-V; clinical + MRI diagnosis; willingness to participate	Not explicitly stated	Pain (VAS); MMO; lateral and protrusive movements; joint sounds; disc position and joint effusion (MRI)	Baseline, weekly after each injection (up to 6 mo), 9 mo	Both i‑PRF and PRP improved pain, MMO, and joint sounds. i‑PRF showed faster and greater improvement in pain and MMO; disc position improved similarly in both groups. Joint effusion decreased more in the i‑PRF group.
Yilmaz 2026 [25]	Turkey	Prospective, 3‑parallel‑arm RCT	Wilkes ≥III internal derangement (MRI); failed conservative Rx; mean age 41 y; 93% female	20	(1) AC alone; (2) AC + I‑PRF injection	AC alone	Wilkes stage ≥3; age 18-65 y; ASA I; MRI within 6 mo; failed conservative treatment	Myofascial pain/cervical pain as primary cause; pregnancy/lactation; systemic joint disease; acute TMJ infection; hematologic disease; prior TMJ surgery; maxillofacial trauma; anti‑inflammatory/muscle relaxant use	Pain (VAS); MMO; joint sounds; OHIP‑14; MFIQ	Baseline, immediate postop, 1 wk, 1 mo, 3 mo	No statistically significant difference between groups at 3 mo. Clinically, i‑PRF showed slightly better pain relief; C‑PRF offered earlier pain and MMO improvements.

**Table 4:** Baseline characteristics of the included patients. AC: arthrocentesis; i‑PRF: injectable platelet‑rich fibrin; C‑PRF: concentrated platelet‑rich fibrin; PRP: platelet‑rich plasma; ARS: anterior repositioning splint; MMO: maximum mouth opening; MIO: maximum interincisal opening; VAS: Visual Analogue Scale; F: female; M: male; R: right; L: left; DDWR: disc displacement with reduction; DDWoR: disc displacement without reduction; OA: osteoarthritis; NR: not reported; NS: not significant; Palp: palpation; Chew: chewing; Mov: jaw movements; Med: median; TMJ-OA: temporomandibular joint osteoarthritis; CBCT: cone-beam computed tomography; MRI: magnetic resonance imaging; DC/TMD: Diagnostic Criteria for Temporomandibular Disorders

Study	Arm	Sample size	Age (years), mean ± SD	Sex (F/M)	Laterality (R/L/Bilateral)	Wilkes stage/diagnosis	Symptom duration (months), mean ± SD	Baseline pain (VAS), mean ± SD	Baseline MIO/MMO (mm), mean ± SD
Chaulagain 2025 [17]	Intervention	16	27.2 ± 8.5	14 F, 2 M	R 9; L 7	II: 1; III: 12; IV: 3	NR	7.4 ± 0.9	25.1 ± 3.1 (MIO)
	Control	16	29.9 ± 7.8	11 F, 5 M	R 7; L 9	II: 6; III: 8; IV: 2		6.7 ± 0.9	27.3 ± 3.3 (MIO)
Ghoneim 2022 [18]	Intervention	20	26.45 ± 8.21	16 F, 4 M	NR (unilateral joints)	DDWR (MRI‑confirmed)	NR	6 ± 1.4	31.48 ± 8.52 (MIO)
	Control	20	28.60 ± 8.42	13 F, 7 M	NR (unilateral joints)	DDWR (MRI‑confirmed)		8 ± 1.3	36.15 ± 7.26 (MIO)
Isık 2022 [19]	Intervention	18	44.67 ± 12.13	16 F, 2 M	NR (22 degenerative joints)	TMJ‑OA (DC/TMD + CBCT)	NR	Palp: 7.83 ± 1.2; Chew: 8 ± 1.41; Mov: 7.61 ± 1.09	33.28 ± 1.52 (MMO)
	Control	18	45.72 ± 13.12	17 F, 1 M	NR (21 degenerative joints)	TMJ‑OA (DC/TMD + CBCT)		Palp: 8.06 ± 1.16; Chew: 8.33 ± 1.23; Mov: 7.83 ± 1.15	33.89 ± 0.96 (MMO)
Isık 2023 [20]	Intervention	38	47.2 ± 9.1	34 F, 4 M	L 13; R 20; Bilateral 5	DDWoR (DC/TMD + MRI)	11.9 ± 3.2	Palp: 7.8 ± 1.5; Chew: 8.0 ± 1.3; Mov: 7.7 ± 1.4	31 ± 1.6 (MMO)
	Control	38	46.8 ± 10.2	35 F, 3 M	L 13; R 19; Bilateral 6	DDWoR (DC/TMD + MRI)	12.1 ± 3.5	Palp: 7.9 ± 1.4; Chew: 8.2 ± 1.2; Mov: 7.7 ± 1.3	30.9 ± 1.8 (MMO)
Karadayi 2021 [21]	Intervention	18	39.97 ± 10.5	10 F, 8 M	NR (unilateral)	Wilkes III: 6; IV: 6; V: 6	NR	5.94 ± 1.67	31.67 ± 9.89 (MIO)
	Control	18	39.67 ± 11	9 F, 9 M	NR (unilateral)	Wilkes III: 6; IV: 6; V: 6		6.22 ± 2.63	33.39 ± 11.22 (MIO)
Nasef 2024 [22]	Intervention	15	NR	NR	Bilateral in all	DDWoR (MRI‑confirmed)	NR	7.3 ± 1.3	25.5 ± 4.7 (MMO)
	Control	15			Bilateral in all	DDWoR (MRI‑confirmed)		6.8 ± 1.3	26.7 ± 5.5 (MMO)
Metello Neves 2026 [23]	Intervention	18	52	13 F, 5 M	NR	DDWR: 12; DDWoR: 6	NR	(Joint sound VAS) 5.83 ± 3.7	41.8 ± 6.2 (MMO)
	Control	18		13 F, 5 M		DDWR: 11; DDWoR: 2 (estimated)		(Joint sound VAS) 5.78 ± 2.96	NR
Sharma 2023 [24]	Intervention	7 (14 joints)	20-50	NR	NR (28 joints)	Wilkes I-V (clinical + MRI)	NR	7.07 ± 1.48	27.86 ± 5.24 (MIO)
	Control	7 (14 joints)			NR (28 joints)	Wilkes I-V (clinical + MRI)		7.11 ± 1.1	28.14 ± 3.48 (MIO)
Yilmaz 2026 [25]	Intervention	10	40.70 ± 12.80	10 F, 0 M	NR (unilateral)	Wilkes ≥ III (MRI)	NR	6.6 ± 1.58	36.8 ± 4.96 (MMO)
	Control	10	40.80 ± 11.76	9 F, 1 M	NR (unilateral)	Wilkes ≥ III (MRI)		7.6 ± 1.43	30.6 ± 7.41 (MMO)

**Figure 2:**
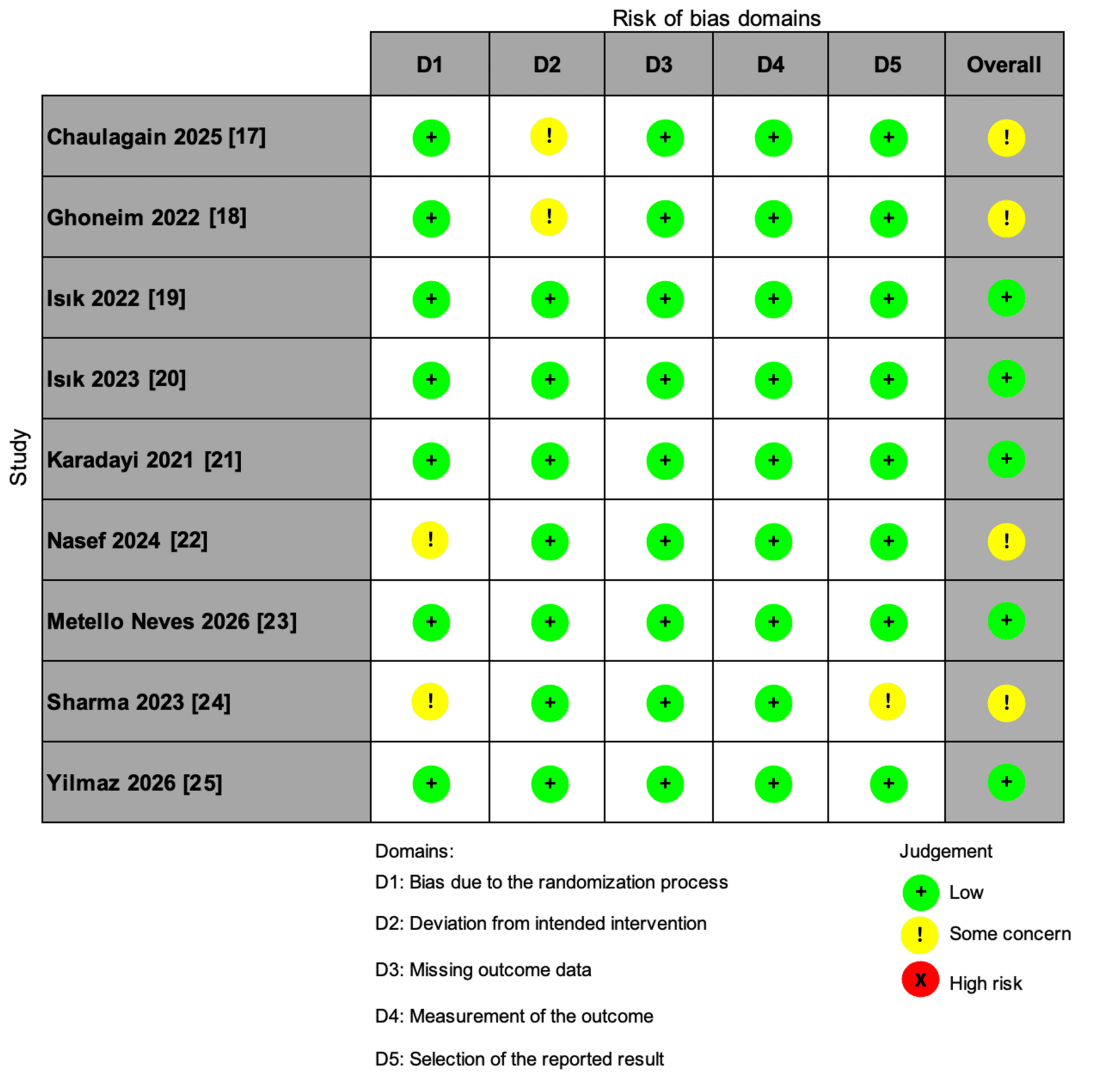
Risk of bias assessment of the included RCTs using the Cochrane ROB-2 tool. RCT: randomized controlled trial; ROB-2: Risk of Bias 2 [17-25]

All RCTs were assessed for risk of bias using the Cochrane ROB-2 tool; five trials had a low risk of bias, while the other four had some concern due to bias arising from the randomization process, deviations from the intended intervention, and selection of reported results (Figure 2).

Primary Outcome

**Figure 3:**
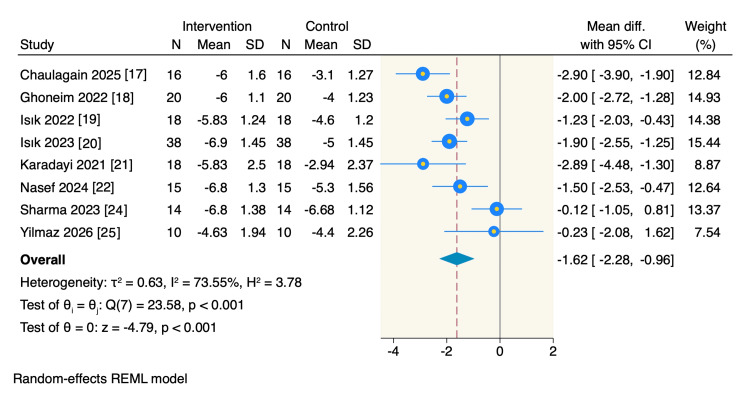
Random-effect model of pain score (VAS) change post-intervention and control. VAS: Visual Analogue Scale; REML: restricted maximum likelihood; CI: confidence interval; SD: standard deviation; diff.: difference [17-22,24,25]

**Figure 4:**
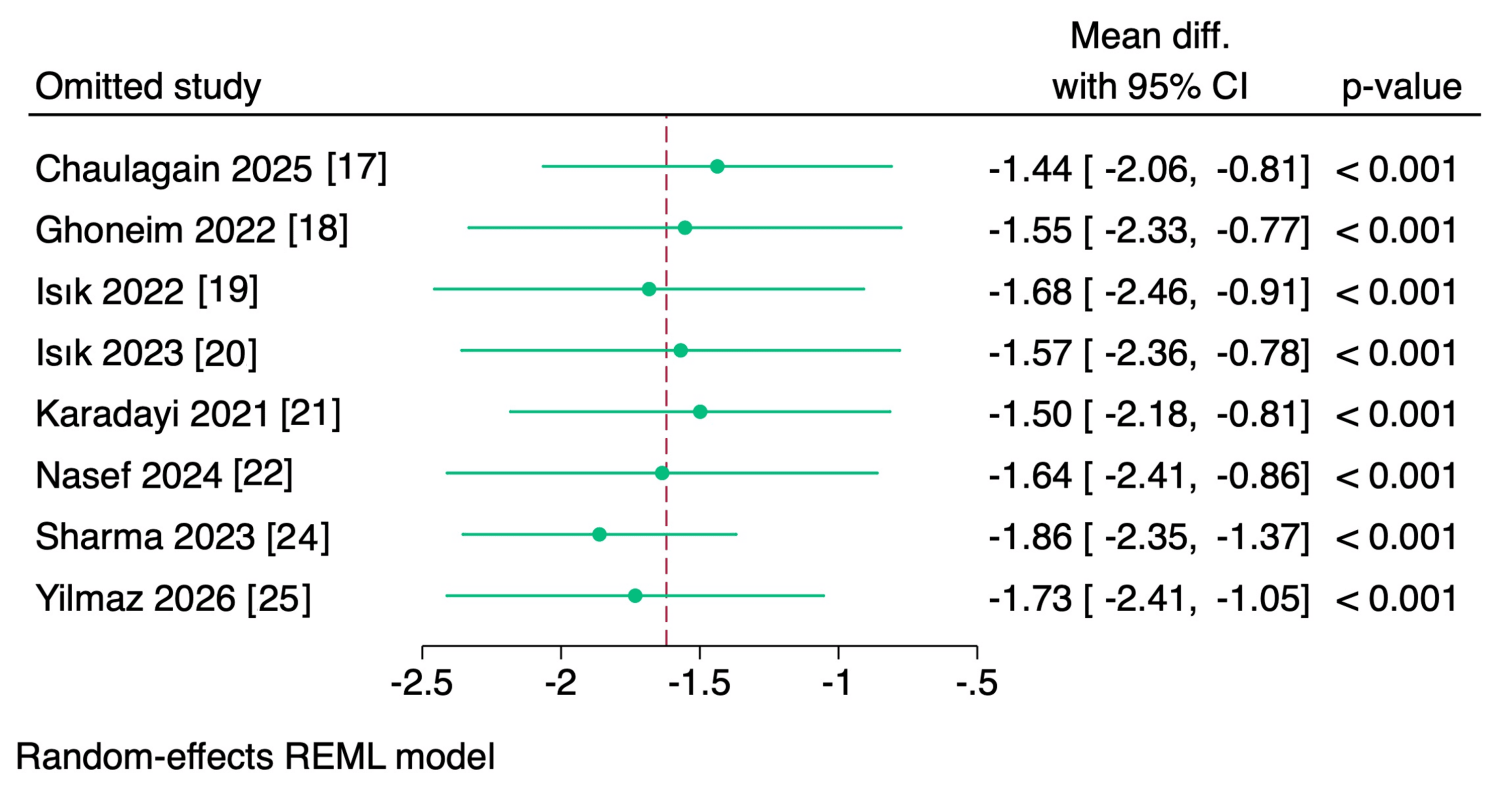
Leave-one-out sensitivity analysis of pain score change. REML: restricted maximum likelihood; CI: confidence interval; diff.: difference [17-22,24,25]

**Figure 5:**
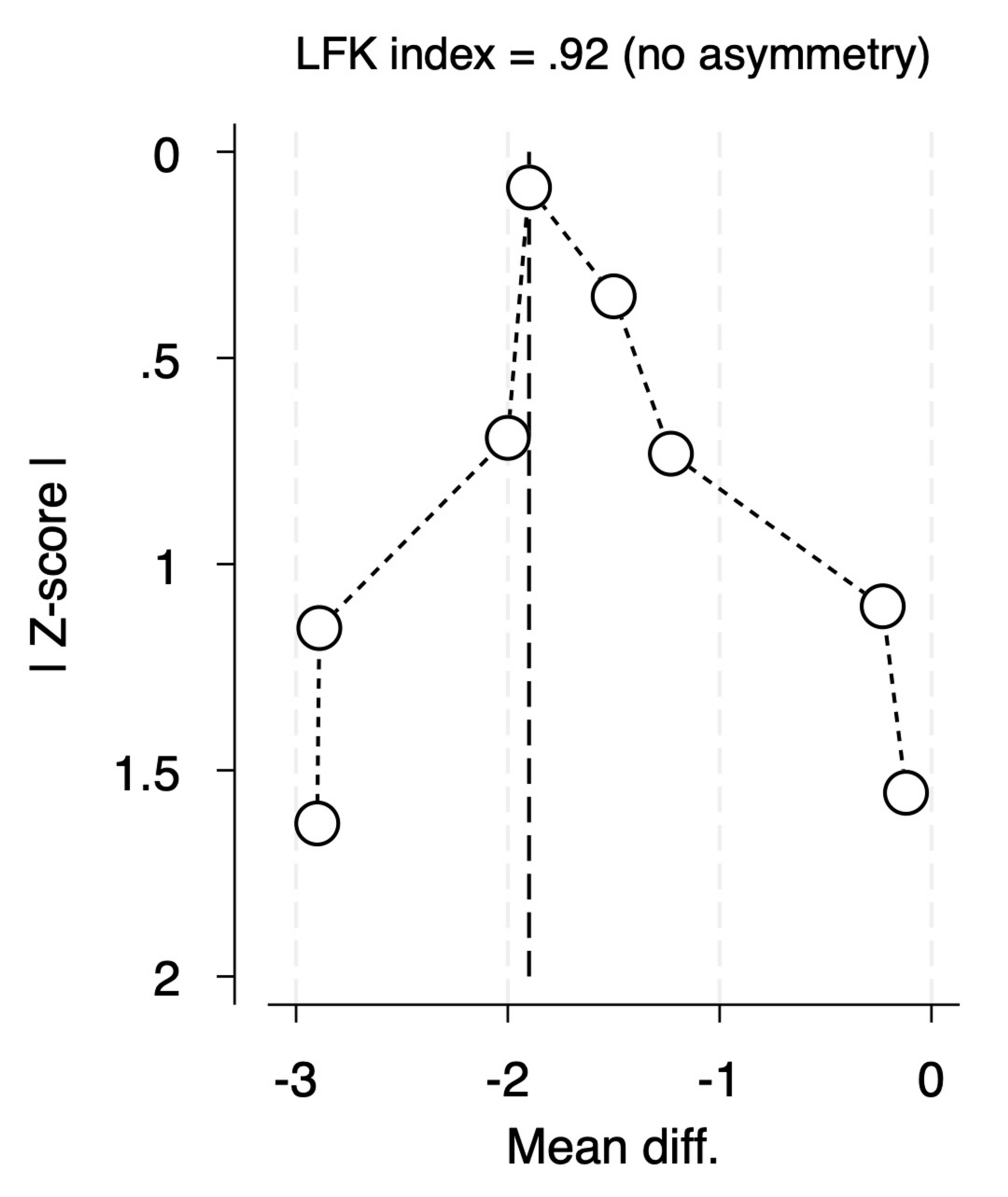
DOI plot with LFK index of pain score change. diff.: difference; DOI: digital object identifier; LFK: Luis Furuya-Kanamori

Eight of the included studies reported a change in pain scores between baseline and follow-up. The pooled estimate demonstrated a significant reduction in pain scores favoring the intervention group compared with the control group (MD -1.62 points, 95% CI: -2.28 to -0.96, p<0.001; I^2^= 73.55%) (Figure 3). We performed a leave-one-out sensitivity analysis, and none of the studies had a disproportional effect on the pooled estimate (Figure 4). The DOI plot showed no asymmetry between the included studies with an LFK index of 0.92 (Figure 5).

**Figure 6:**
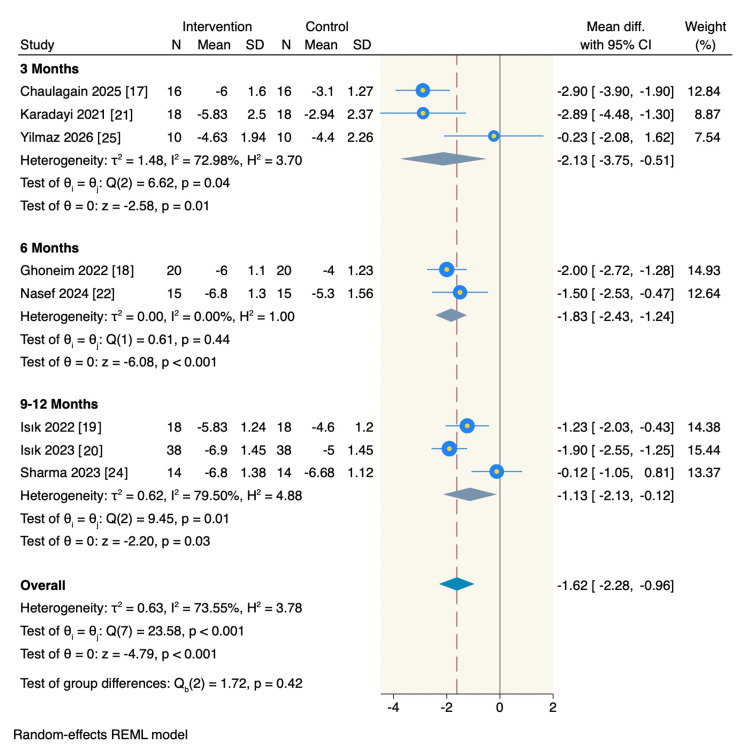
Subgroup analysis of pain score change based on follow-up duration. REML: restricted maximum likelihood; CI: confidence interval; SD: standard deviation; diff.: difference [17-22,24,25]

The subgroup analysis based on follow-up duration showed a significant reduction in pain score across all follow-up periods (at three months: MD -2.13 points (95% CI: -3.75, -0.51); at six months: MD -1.83 points (95% CI: -2.43, -1.24), and between 9-12 months: MD -1.13 points (95% CI: -2.13, -0.12)) (Figure 6). 

**Figure 7:**
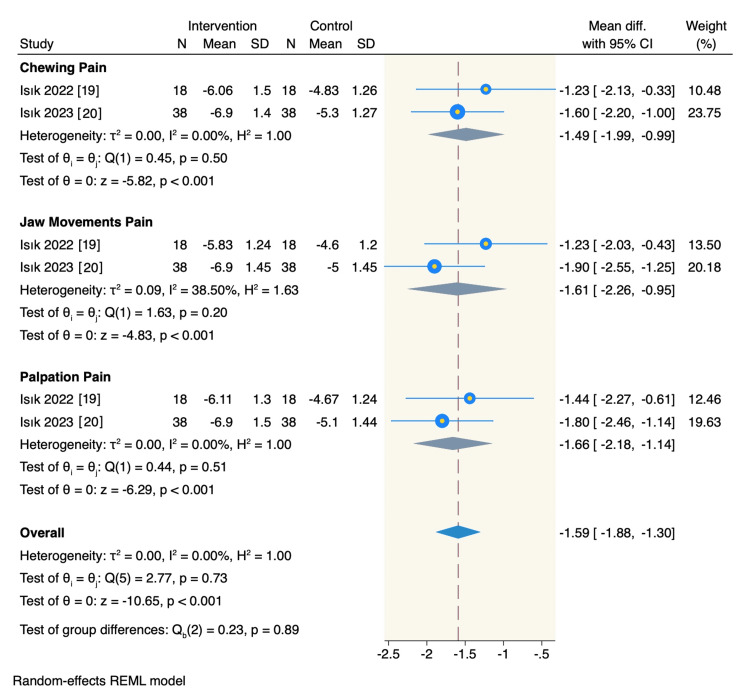
Random-effect model of type of pain score changes (exploratory analysis). REML: restricted maximum likelihood; CI: confidence interval; SD: standard deviation; diff.: difference [19,20]

Additionally, the exploratory subgroup analysis according to pain type showed a significant reduction in VAS score in all three types (pain when chewing: MD -1.49 points (95% CI: -1.99, -0.99); pain during jaw movements: MD -1.61 points (95% CI: -2.26, -0.95), and palpation pain: MD -1.66 points (95% CI: -2.18, -1.14)) (Figure 7). 

**Figure 8:**
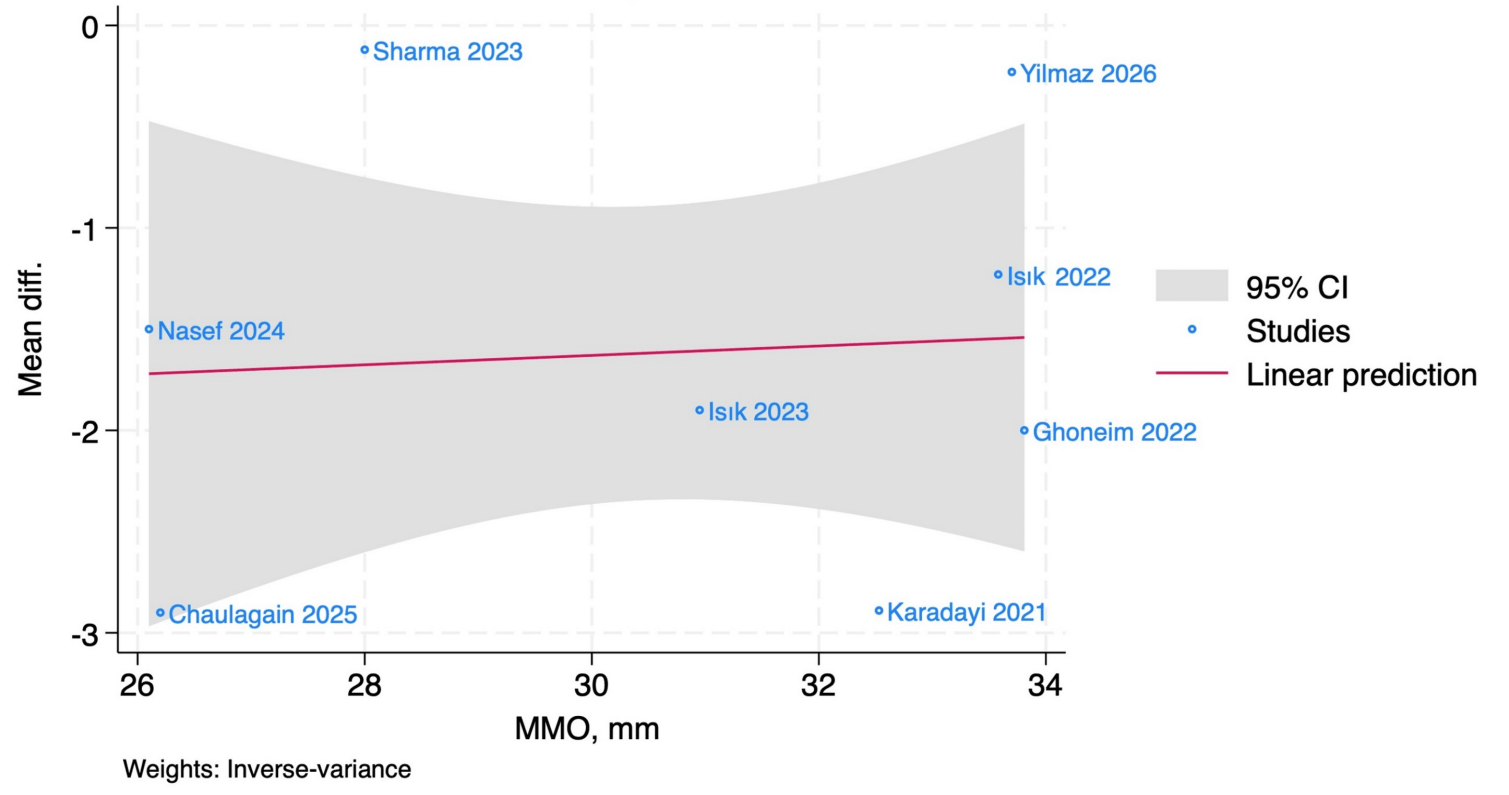
Bubble plot of random-effect meta-regression of pain score change. diff.: difference; MMO: maximum mouth opening; CI: confidence interval [17-22,24,25]

The random-effects meta-regression showed no significant effect of baseline MMO over the change in pain scores (coefficient: 0.0232 (95% CI: -0.2078, 0.2542), p= 0.844) (Figure 8). 

**Figure 9:**
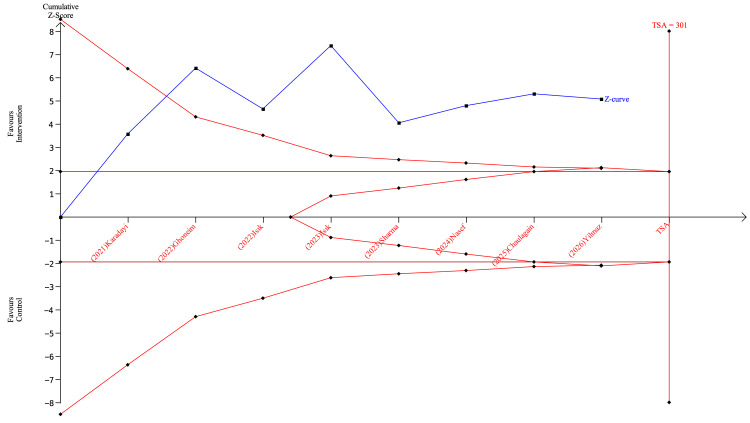
TSA of pain score outcome. TSA: trial sequential analysis [17-22,24,25]

The TSA showed that the Z-curve crossed both the conventional boundary and the sequence monitoring boundary, confirming the conclusive and sufficient confidence levels of the results (Figure 9). 

Secondary Outcomes

**Figure 10:**
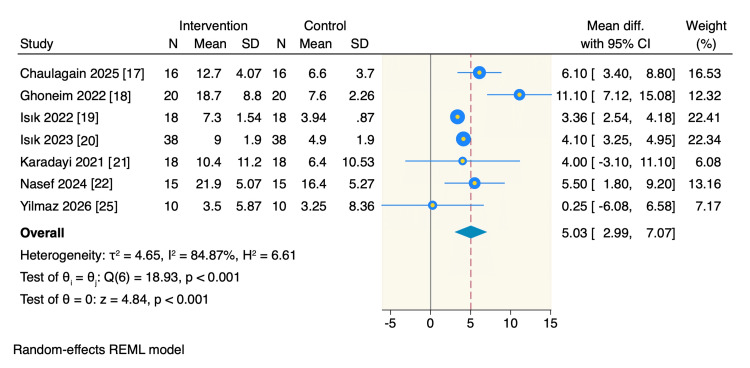
Random-effect model of MMO change. REML: restricted maximum likelihood; CI: confidence interval; SD: standard deviation; diff.: difference; MMO: maximum mouth opening [17-22,25]

The change in MMO was reported by seven studies, of which the pooled estimate showed a greater mouth opening between the patients in the intervention group by 5.03 mm compared with the control group (MD 5.03 mm, 95% CI: 2.99 to 7.07, p<0.001; I^2^=84.87) (Figure 10). 

**Figure 11:**
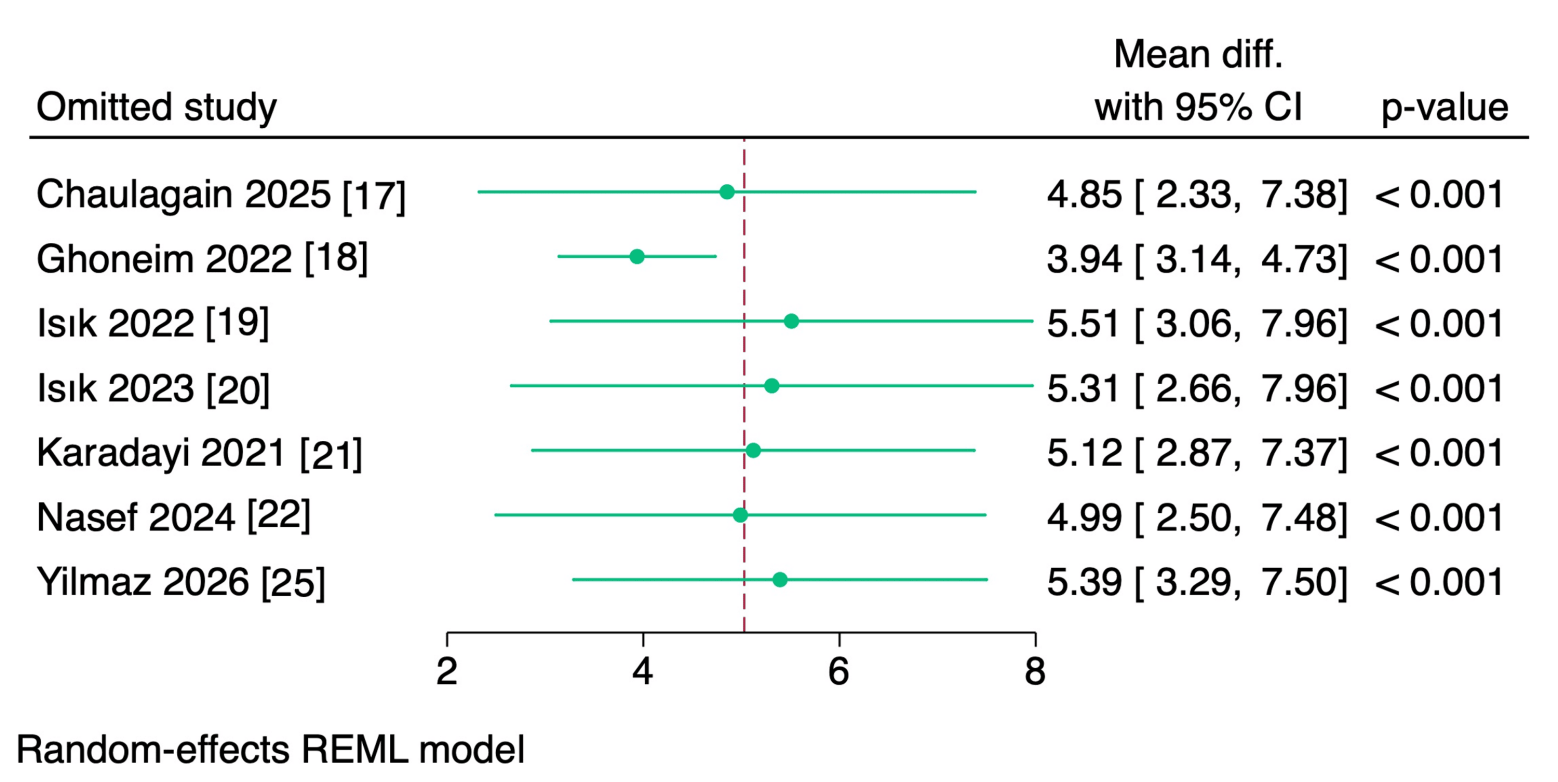
Leave-one-sensitivity analysis of MMO. REML: restricted maximum likelihood; CI: confidence interval; diff.: difference; MMO: maximum mouth opening [17-22,25]

The leave-one-out sensitivity analysis showed none of the included studies had a disproportional effect on the pooled estimate (Figure 11). 

**Figure 12:**
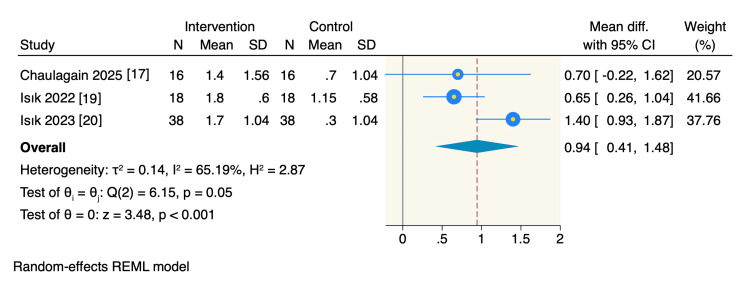
Random-effect model of contralateral excursion change. REML: restricted maximum likelihood; CI: confidence interval; SD: standard deviation; diff.: difference [17,19,20]

**Figure 13:**
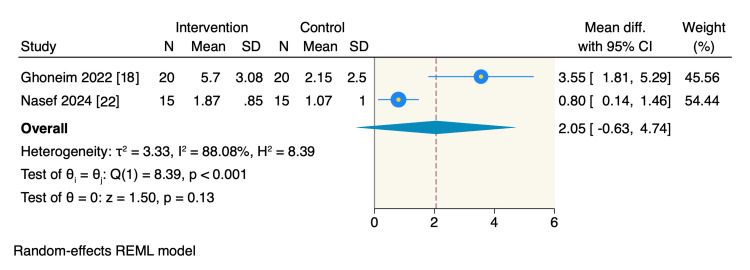
Random-effect model of right lateral excursion change. REML: restricted maximum likelihood; CI: confidence interval; SD: standard deviation; diff.: difference [18,22]

**Figure 14:**
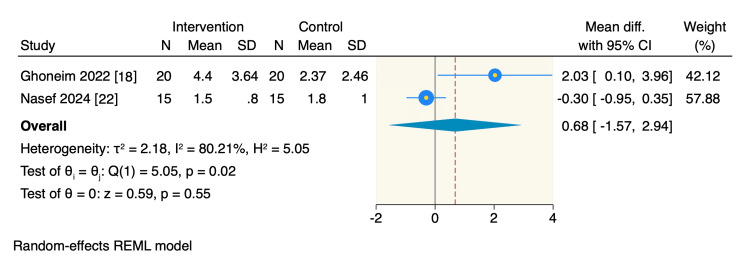
Random-effect model of left lateral excursion change. REML: restricted maximum likelihood; CI: confidence interval; SD: standard deviation; diff.: difference [18,22]

The pooled estimate showed a significant increase in contralateral excursion by 0.94 mm in the intervention group (MD 0.94 mm, 95% CI: 0.41 to 1.48, p<0.001) (Figure 12), with no significant difference in the right (MD 2.05 mm, 95% CI: -0.63 to 4.74, p=0.13) (Figure 13) or the left lateral excursion (MD 0.68 mm, 95% CI: -1.57 to 2.94, p= 0.55) (Figure 14). 

**Figure 15:**
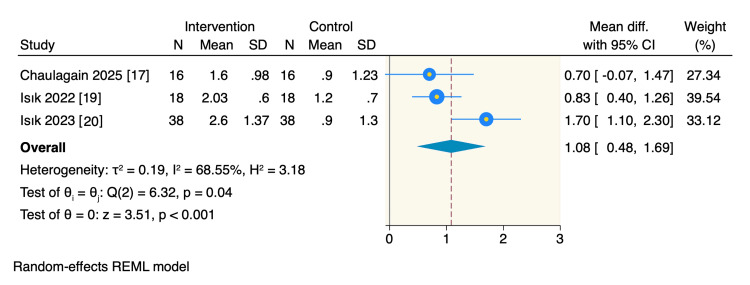
Random-effect model of protrusive movement change. REML: restricted maximum likelihood; CI: confidence interval; SD: standard deviation; diff.: difference [17,19,20]

**Figure 16:**
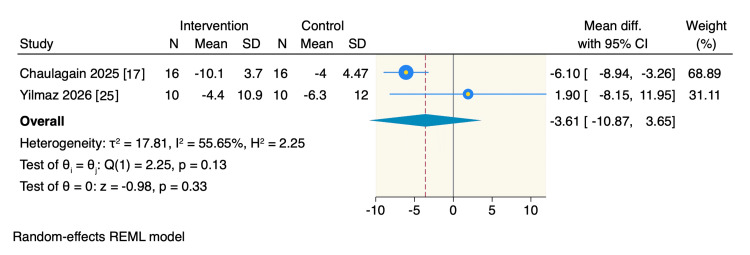
Random-effect model of QoL change. REML: restricted maximum likelihood; CI: confidence interval; SD: standard deviation; diff.: difference; QoL: quality of life [17,25]

Additionally, the intervention increased the protrusive movement by 1.08 mm compared with the control (MD 1.08 mm, 95% CI: 0.48 to 1.69, p<0.001) (Figure 15), with no significant difference in overall QoL (MD -3.61 points, 95% CI: -10.87 to 3.65, p=0.33) (Figure 16). 

GRADE Assessment

As shown in Table 5, the strength of evidence was moderate in all studied clinical outcomes. 

**Table 5:** GRADE assessment. ⨁⨁⨁◯ indicates moderate certainty of the evidence based on the GRADE scale. ^a ^indicates that a significant heterogeneity was detected. CI: confidence interval; MD: mean difference; VAS: Visual Analogue Scale; OHIP-14: Oral Health Impact Profile-14; GRADE: Grading of Recommendations Assessment, Development, and Evaluation [17-25]

Certainty assessment							No. of patients		Effect		Certainty	Importance
No. of studies	Study design	Risk of bias	Inconsistency	Indirectness	Imprecision	Other considerations	Intervention	Control	Relative (95% CI)	Absolute (95% CI)		
Pain (follow-up: range 3 months to 12 months; assessed with: MD; scale (VAS) from: 1 to 10)												
8	Randomized trials	Not serious	Not serious	Not serious	Serious^a^	None	149	149	-	MD 1.62 points lower (2.28 lower to 0.96 lower)	⨁⨁⨁◯ Moderate^a^	CRITICAL
Maximum mouth opening (follow-up: range 3 months to 12 months; assessed with: MD)												
7	Randomized trials	Not serious	Not serious	Not serious	Serious^a^	None	135	135	-	MD 5.03 mm higher (2.99 higher to 7.07 higher)	⨁⨁⨁◯ Moderate^a^	CRITICAL
Contralateral excursion (follow-up: range 3 months to 12 months; assessed with: MD)												
3	Randomized trials	Not serious	Not serious	Not serious	Serious^a^	None	72	72	-	MD 0.94 mm higher (0.41 higher to 1.48 higher)	⨁⨁⨁◯ Moderate^a^	IMPORTANT
Right lateral excursion (follow-up: range 3 months to 12 months; assessed with: MD)												
2	Randomized trials	Not serious	Not serious	Not serious	Serious^a^	None	35	35	-	MD 2.05 mm higher (0.63 lower to 4.74 higher)	⨁⨁⨁◯ Moderate^a^	IMPORTANT
Left lateral excursion (follow-up: range 3 months to 12 months; assessed with: MD)												
2	Randomized trials	Not serious	Not serious	Not serious	Serious^a^	None	35	35	-	MD 0.68 mm higher (1.57 lower to 2.94 higher)	⨁⨁⨁◯ Moderate^a^	IMPORTANT
Protrusive movement (follow-up: range 3 months to 12 months; assessed with: MD)												
3	Randomized trials	Not serious	Not serious	Not serious	Serious^a^	None	72	72	-	MD 1.08 mm higher (0.48 higher to 1.69 higher)	⨁⨁⨁◯ Moderate^a^	IMPORTANT
Quality of life (follow-up: range 3 months to 12 months; assessed with: MD; scale (OHIP‑14) from: 0 to 56)												
2	Randomized trials	Not serious	Not serious	Not serious	Serious^a^	None	26	26	-	MD 3.61 OHIP-14 lower (10.87 lower to 3.65 higher)	⨁⨁⨁◯ Moderate^a^	IMPORTANT

Discussion

The present updated meta-analysis demonstrates that arthrocentesis combined with i-PRF is associated with significant improvement in pain reduction and mandibular function in patients with TMD compared with control interventions. Patients receiving i-PRF showed lower VAS pain scores and greater MMO, in addition to improved contralateral excursion and protrusive movement. Importantly, the beneficial effect on pain reduction remained significant across all follow-up periods. Moreover, the TSA findings confirmed that the available evidence for the primary outcome was conclusive and sufficiently powered, supporting the robustness of the pooled estimates.

The favorable outcomes observed with i-PRF may be explained by its regenerative and anti-inflammatory properties [26]. i-PRF contains a high concentration of platelets, leukocytes, fibrin matrix, and multiple growth factors that may enhance tissue healing and modulate intra-articular inflammation [27]. In the setting of TMJ disorders, intra-articular administration of i-PRF following arthrocentesis may improve synovial tissue repair, reduce inflammatory mediators, and promote restoration of joint function [28,29]. Additionally, the fibrin scaffold of i-PRF allows gradual release of biologically active mediators, which may contribute to sustained clinical improvement over time [30].

Our findings are generally consistent with previously published studies evaluating biologic adjuncts in TMJ arthrocentesis [21,22,31]. However, prior evidence was largely limited by smaller sample sizes, the absence of comprehensive subgroup analyses, and the lack of TSA [31]. The present study provides an updated synthesis of the currently available randomized evidence and further strengthens the evidence supporting the use of i-PRF as an adjunctive therapy in symptomatic TMD patients.

Despite the significant pooled effects, moderate-to-high heterogeneity was observed in several outcomes. This variability may be attributed to differences in patient populations, Wilkes classification stages, lavage protocols, centrifugation methods, injection techniques, and follow-up durations across the included studies [18,23]. Nevertheless, leave-one-out sensitivity analyses demonstrated that no individual study had a disproportionate influence on the overall pooled estimates, suggesting that the findings remained statistically robust despite the observed heterogeneity.

Clinical Relevance

From a clinical perspective, the present findings support the growing role of biologic adjunctive therapies in minimally invasive TMJ management. Arthrocentesis alone is effective in reducing intra-articular pressure and removing inflammatory mediators; however, residual symptoms and recurrence may still occur in some patients [32,33]. The addition of i-PRF may therefore provide a dual therapeutic effect through both mechanical lavage and biologic enhancement of tissue healing [34,35]. Given its relatively simple preparation, autologous nature, and minimally invasive application, i-PRF may represent a practical adjunctive option in patients with persistent symptomatic TMD who do not adequately respond to conservative therapy alone.

Strengths and Limitations

The present study has several strengths. First, only RCTs were included, improving the overall quality of the synthesized evidence. Second, we performed multiple complementary analyses, including subgroup analysis, sensitivity analysis, meta-regression, DOI plot assessment, TSA, and GRADE evaluation. Third, the consistency of the findings across different follow-up periods supports the reproducibility of the observed treatment effects.

Several limitations should also be acknowledged. The included studies had relatively small sample sizes and short-to-intermediate follow-up durations. Additionally, substantial heterogeneity was present in some analyses due to variations in study protocols and patient characteristics. Some secondary outcomes, particularly QoL measures, were reported by a limited number of studies, reducing the precision of these estimates. Moreover, small study effects and possible publication bias could not be ruled out. Finally, the absence of patient-level data limited the ability to perform detailed subgroup analyses according to disease severity or procedural characteristics.

## Conclusions

Arthrocentesis combined with i-PRF appears to provide significant improvement in pain reduction and mandibular functional outcomes in patients with TMD compared with control interventions. The available evidence suggests that i-PRF may represent a promising adjunctive therapy following arthrocentesis. However, larger randomized trials with longer follow-up durations are still needed to confirm the long-term clinical benefits and establish standardized treatment protocols.
